# *In Vivo* Expression of Reprogramming Factor OCT4 Ameliorates Myelination Deficits and Induces Striatal Neuroprotection in Huntington’s Disease

**DOI:** 10.3390/genes12050712

**Published:** 2021-05-10

**Authors:** Ji-Hea Yu, Bae-Geun Nam, Min-Gi Kim, Soonil Pyo, Jung-Hwa Seo, Sung-Rae Cho

**Affiliations:** 1 Department and Research Institute of Rehabilitation Medicine, Yonsei University College of Medicine, Seoul 03722, Korea; onlyjin112@yuhs.ac (J.-H.Y.); nbg6824@naver.com (B.-G.N.); neuro94@naver.com (S.P.); zugula@naver.com (J.-H.S.); 2 Brain Korea 21 PLUS Project for Medical Science, Yonsei University, Seoul 03722, Korea; mg0521k@naver.com; 3 Graduate Program of Nano Science and Technology, Yonsei University College of Medicine, Seoul 03722, Korea; 4 Rehabilitation Institute of Neuromuscular Disease, Yonsei University College of Medicine, Seoul 03722, Korea

**Keywords:** Huntington’s disease, reprogramming, octamer-binding transcription factor 4, oligodendrocyte progenitor cells

## Abstract

White matter atrophy has been shown to precede the massive loss of striatal GABAergic neurons in Huntington’s disease (HD). This study investigated the effects of *in vivo* expression of reprogramming factor octamer-binding transcription factor 4 (OCT4) on neural stem cell (NSC) niche activation in the subventricular zone (SVZ) and induction of cell fate specific to the microenvironment of HD. R6/2 mice randomly received adeno-associated virus 9 (AAV9)-OCT4, AAV9-Null, or phosphate-buffered saline into both lateral ventricles at 4 weeks of age. The AAV9-OCT4 group displayed significantly improved behavioral performance compared to the control groups. Following AAV9-OCT4 treatment, the number of newly generated NSCs and oligodendrocyte progenitor cells (OPCs) significantly increased in the SVZ, and the expression of OPC-related genes and glial cell-derived neurotrophic factor (GDNF) significantly increased. Further, amelioration of myelination deficits in the corpus callosum was observed through electron microscopy and magnetic resonance imaging, and striatal DARPP32^+^ GABAergic neurons significantly increased in the AAV9-OCT4 group. These results suggest that *in situ* expression of the reprogramming factor OCT4 in the SVZ induces OPC proliferation, thereby attenuating myelination deficits. Particularly, GDNF released by OPCs seems to induce striatal neuroprotection in HD, which explains the behavioral improvement in R6/2 mice overexpressing OCT4.

## 1. Introduction

Huntington’s disease (HD) pathology caused by an expansion of the cytosine-adenine-guanine (CAG) repeats within the HTT gene is characterized by massive loss of neurons in striatum and deep layers of the cortex as well as early and progressive thinning of white matter (WM) [1]. WM changes can be observed in the striatum nearby the corpus callosum (CC), and in the posterior WM tracts at a pre-symptomatic (pre-HD) stage [1,2,3,4,5,6].

Although the mechanism underlying abnormalities of WM was unclear, neuroimaging data supported the hypothesis that myelin breakdown leads to WM atrophy in human and mouse models of HD [7,8]. Interestingly, an increase in the density of oligodendrocytes in the striatum of pre-HD patients was demonstrated before striatal degeneration as a compensatory neuroprotective response [9,10,11].

Previous studies have shown that the manifestation of clinical symptoms such as motor defects in HD is closely associated with early changes in WM with demyelination [5,12,13,14], suggesting that targeting oligodendrocyte could be therapeutic in HD. A recent study reported that reduction of the mHTT in oligodendrocytes prevented behavioral deficits and demyelination in HD mice [15]. However, chronic demyelination in HD at late-symptomatic (late-HD) stage can be occurred by the malfunction of oligodendrocytes and myelinating repair system [16].

Under demyelinating conditions including pre-HD stage, it can induce proliferation of neural stem cells (NSCs) and neural progenitors [17], give rise to oligodendrocyte progenitor cells (OPCs) [18], and then differentiate into oligodendrocytes [11]. An increase in the number of OPCs derived from NPCs under demyelination contributed the generation of oligodendrocyte. However, these cells do not necessarily contribute to chronic diseases with extensive myelin loss [19,20,21,22].

*In vivo* reprogramming toward a plastic state has emerged as a new approach for treating neurological diseases [23,24]. Representative reprogramming factors, octamer-binding protein 4 (OCT4), sex determining region Y-box 2 (SOX2), Krüppel-like factor 4 (KLF4) and MYC, can manipulate cell fate [25]. Among these factors, these factors can be replaced with others, and OCT4 remained essential [26,27]. Previous studies revealed that the ectopic expression of OCT4, a key reprograming factor, is sufficient to directly reprogram NSCs into a pluripotent and plastic state [28,29] and allowed differentiation into neurodegenerative disease-specific environment. In addition, OCT4-induced reprogramming increases NSC proliferation in the (SVZ) of adult brain. The stimulation of endogenous NSCs is a useful tool for a therapeutic approach in neurodegenerative disorders including HD [30,31,32] and demyelinated condition such as spinal cord injury [33]. Moreover, previous studies suggest that the expression of OCT4 is absent in adult mice [34,35].

Therefore, our hypothesis is that the OCT4 overexpression in pre-HD condition at 4 weeks of age can induce NSC niche activation in the SVZ and induction of cell fate specific to the changed microenvironment of HD. Finally, we found that the *in situ* expression of OCT4 in the SVZ induces OPC proliferation, thereby attenuating myelination deficits. Additionally, myelin regulatory factor (MYRF) and glial cell-derived neuroprotective factor (GDNF) released by OPCs induced striatal neuroprotection in HD, which can explain the behavioral improvement in R6/2 mice overexpressing OCT4.

## 2. Materials and Methods

### 2.1. Mice

R6/2 strain, a transgenic mouse model of HD carrying approximately 160 ± 5 CAG repeats, was obtained from the Jackson Laboratory (B6CBA-Tg (HDexon1) 62Gpb/1J, Stock No: 002810). These transgenic mice mimic human HD with many neurological phenotypes, including choreiform-like movements, involuntary stereotypic movements, tremor, epileptic seizures, and non-movement disorder components including unusual vocalization. The symptoms of R6/2 mice become apparent between 6 and 8 weeks of age and typically die nearby 13 weeks of age [36,37,38]. To prevent dehydration and malnutrition at the terminal stage of animals, we daily provided water-soaked food pellets. All animals were housed in a facility accredited by the Association for Assessment and Accreditation of Laboratory Animal Care (AAALAC). Experimental procedures were approved by the Institutional Animal Care and Use Committee (IACUC 2016-0298, 2020-0007). The mice were kept in a temperature-controlled room on a 12-h light/dark cycle and food and water ad libitum.

**Figure 1:**
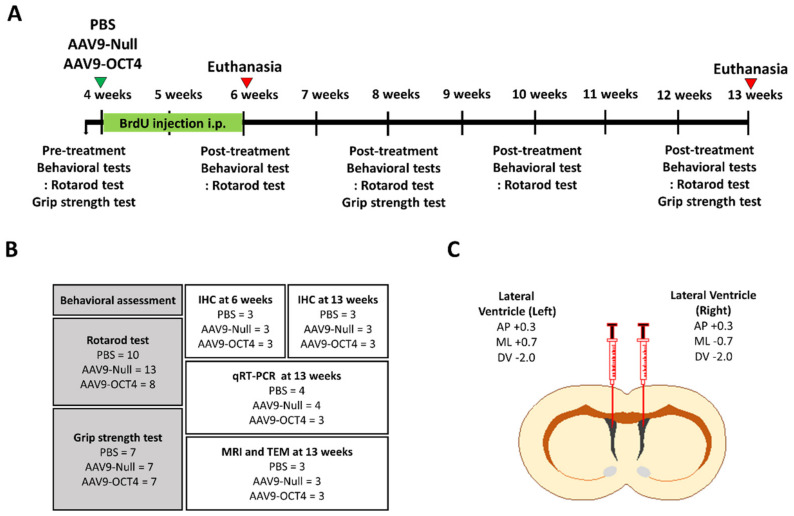
Experimental scheme for *in vivo* expression of OCT4 in HD mice. (**A**) The schedule of OCT4 treatment and behavioral tests (rotarod and grip strength tests) in the timeline are presented. (**B**) A total of thirty-one R6/2 mice were randomly assigned to PBS (N= 10), AAV9-Null (N = 13) and AAV9-OCT4 (N = 8) for behavioral assessment to investigate behavioral outcomes. Among the subjects, nine mice were recruited for the IHC, MRI and TEM imaging at 13 weeks of age (N = 3 per group). A total of eleven mice were also recruited to evaluate OPC related genes using a qRT-PCR (PBS (N = 4), AAV9-Null (N = 4), AAV9-OCT4 (N = 3). (**C**) Both side lateral ventricle injection using stereotaxic surgery performed at 4 weeks of age.

A schematic timeline of this experiment during the nine weeks is provided in Figure 1A. For behavioral assessments until 13 weeks, thirty-one male and female R6/2 mice (phosphate-buffered saline (PBS) (N = 10), Adeno-associated virus 9 (AAV9-Null) (N = 13), AAV9-OCT4 (N = 8)) were used. In addition, for immunostaining until 6 and 13 weeks, nine male and female mice were used (N = 3 per group). Among the subjects, mice were also recruited for qPCR at 13 weeks of age (PBS (N = 4), AAV9-Null (N = 4), AAV9-OCT4 (N = 3)). The mice were randomly assigned to either PBS, AAV9-Null, AAV9-OCT4 (N = 3 per group) to confirm demyelination via MRI and TEM (Figure 1B).

### 2.2. AAV9 Viral Vector Stereotaxic Injection

At 4 weeks of age, mice were anesthetized with intraperitoneal (IP) injection of ketamine (100 mg/kg; Huons, Gyeonggi-do, Korea) and xylazine (10 mg/kg; Bayer Korea, Seoul, Korea). A stereotaxic procedure was performed at 4 weeks of age in which the mice received both lateral ventricle (LV) injections (1 × 10^12^ vg/mL, 1 μL each) using the following stereotaxic coordinates: AP +0.3 mm from bregma, ML +0.7/−0.7 mm from bregma, and DV −2.0 mm from dura mater (Figure 1C). AAV9 vector (ViroVek, Hayward, CA, USA) containing human OCT4-HA tag was expressed using the CMV promotor (Figure S1A). Mice were randomly assigned to one of the following groups: PBS, AAV9-Null, or AAV9-OCT4 treatment.

### 2.3. Rotarod Test

A rotarod test (No.47600, Ugo Basile, Comerio, Italy) was used to assess motor coordination and locomotor function at 4, 6, 8, 10, and 13 weeks of age. The rolling rod was set to an accelerating speed (4–40 rpm) and a constant speed (12 and 16 rpm), and the latency fall time was measured [39]. An individual test was terminated at a maximum latency of 300 s if the mouse did not fall.

In order to prevent motor learning of the mice, we conducted two minutes of adaptation time at 4 rpm and took additional trials for adaptation before recorded. We also took resting time more than half hour whenever the speed of the rotarod test changes.

### 2.4. Grip Strength Test

A grip strength test was performed using the SDI Grip Strength System (San Diego Instruments, Inc., San Diego, CA, USA), which includes a push-pull strain gauge at 4, 8 and 12 weeks of age. A 2-mm diameter triangular piece of metal wire was used as the grip bar. Each animal was held near the base of its tail by a researcher and allowed to approach the bar until it was able to grip it with its forepaw. Peak grip force was automatically recorded in kilogram-force (kgf) by the apparatus. The average of peak forces from the three trials was used for the final analysis [39]. Grip force data from the grip strength test were normalized with respect to body weight [40].

### 2.5. Immunohistochemistry (IHC)

Mice were daily given an IP injection of 5-bromo-2′-deoxyuridine (BrdU; 50 mg/kg, Sigma-Aldrich, St. Louis, MO, USA) for 12 days, beginning after stereotaxic surgery [14]. R6/2 mice euthanasia performed at 6 weeks (early-HD stage) and 13 weeks of age (late-HD stage) by transcardial perfusion with cold 1X PBS, followed by 4% paraformaldehyde (PFA). Harvested brain tissues were cryosectioned 16-μm thick slices, and immunohistochemical staining was performed on four sections, representing a range of more than 128 μm. The tissue sections were stained with the following antibodies: cell proliferation marker; BrdU (1:200, abcam, ab6326) and Ki67 (1:400, Leica Biosystems, NCL-Ki67p); OCT4 tagging marker HA (1:400, CSF, 3724S); OCT4 (1:100, santacruz, sc-5279); neuron-specific class III β-tubulin (βIII-tubulin, 1:400, abcam, ab18207) and mature neuronal marker NeuN (1:400, Millipore, MAB377); glial fibrillary acidic protein (GFAP, 1:400, abcam, ab10062) and s100β (1:400, Sigma, S2532); Nestin (1:400, abcam, ab6142); neural/glial antigen 2 (NG2, 1:200, Millipore, ab5320) and dopamine- and cAMP-regulated neuronal phosphoprotein (DARPP-32, 1:400, cell signaling technology, 2306). The stained sections were observed by confocal microscopy (LSM700, Zeiss, Gottingen, Germany) and analyzed using ZEN black and blue edition (Zeiss, Gottingen, Germany).

### 2.6. Real-Time Quantitative Reverse Transcription PCR (qRT-PCR)

At 13 weeks of age, R6/2 mice euthanasia performed for biochemical study and cardiac perfused with cold 1X PBS. Total RNA was extracted from the cortex and the striatum using TRIzol (Invitrogen Life Technologies, Carlsbad, CA, USA). Purified total RNA (1 µg) was used as a template to generate the cDNA using the ReverTra Ace qPCR RT master mix with gDNA remover (TOYOBO). The standard protocol for the qRT-PCR with SYBR Green was provided from roche applied science. A total volume of 20 μL master mix with 1 μL of cDNA was used in the qRT-PCR reaction, which was performed in triplicate on a LightCycler 480 using the LightCycler 480 SYBR Green master mix (Roche Applied Science, Mannheim, Germany). The mRNA abundance of target genes was assayed by qRT-PCR. Glyceraldehyde 3-phosphate dehydrogenase (GAPDH) was used as an internal control. Primers used for qRT-PCR were as follow: platelet-derived growth factor receptor α (PDGFRα), 5′-GGAGACTCAAGTAACCTTGCAC-3′ and 5′-TCAGTTCTGACGTTGCT TTCAA-3′, oligodendrocyte transcription factor 2 (Olig2), 5′-TCCCCAGAACCC GATGATCTT-3′ and 5′-CGTGGACGAGGACACAGTC-3′, NG2, 5′-GGCTTGTGCTG TTCTCACA-3′ and 5′-CACAGACTCTGGACAGACGG-3′, Wnt family member 3 (WNT3), 5′-TAAAGTGTAAATGCCACGGGTT-3′ and 5′-CGGAGGCACTGTCGTACTTG-3′, MYRF, 5′-TCTGGGCCTCCCATCAAAG-3′ and 5′-CGGGGTTATGGTGCGTAGAAG-3′, GDNF, 5′-GCCGGACGGGACTCTAAGAT-3′ and 5′- CGTCATCAAACT GGTCAGGATAA-3′, GFAP, 5′-CGGAGACGCATCACCTCTG-3′ and 5′-AGGGAGTGGAGGAGTCATTCG-3′, βIII-tubulin, 5′-CGCACGACATCTAGGACTGA-3′ and 5′-TGAGGCCTC CTCTCACAAGT-3′, NeuN, 5′-CCACCACTCTCTTGTCCGTT-3′ and 5′-ATCAGCAG CGGCATAGACTC-3′, GAD67, 5′-CTCAGGCTGTATGTCAGATGTTC-3′ and 5′-AAG CGAGTCACAGAGATTGGTC-3′, DARPP32, 5′-AGATTCAGTTCTCTGTGCCCG-3′ and 5′-TGGGTCTCTTCGACTTTGGG-3′ and GAPDH was used as the internal control [3,9]. Primers of GAPDH were 5′-GTCGGTGTGAACGGATTTG-3′ and 5′-GAACATGTAGACCATGTAGTTG-3′. The expression of each gene of interest was obtained using the 2^−ΔΔCt^ method. All results were expressed as means ± standard error of the mean from at least three independent experiments.

### 2.7. Transmission Electron Microscopy (TEM)

For TEM study, mice were perfused and fixed for 12 h in 0.1M phosphate buffer (PB) followed by 4% PFA containing 2% glutaraldehyde (MERCK, ZC814139734) at 13 weeks of age. They were postfixed with 1% osmium tetroxide dissolved in 0.1 M PB for 2 h and dehydrated inascending gradual series (50–100%) of ethanol and infiltrated with propylene oxide. Specimens were embedded by Poly/Bed 812 kit (Polysciences). After pure fresh resin embedding and polymerization at 65 °C electron microscope oven (TD-700, DOSAKA, Japan) for 24 h. Sections of about 200~250 nm thick section were initially cut and stained with toluidine blue (sigma, T3260) for light microscope. Ultra-thin slices (70 nm) were double stained with 6% uranyl acetate (EMS, 22,400 for 20 min) and lead citrate (fisher, for 10 min) for contrast staining. There sections were cut by LEICA EM UC-7 (Leica Microsystems, Austria) with a diamond knife (Diatome) and transferred on copper and nickel grids. All of the thin sections were observed by transmission electron microscopy (JEM-1011, JEOL, Tokyo, Japan) at the acceleration voltage of 80 kV. For analysis of TEM images, we used gRatio version 3 program in MATALAB the watershed and connectivity theorems to calculate the average g-ratio and the diameter distribution.

### 2.8. Magnetic Resonance Imaging (MRI)

At 13 weeks of age, R6/2 mice were anaesthetised with 1–2% isoflurane. MRI experiments were performed with a 9.4 T Bruker Biospec scanner (Ettlingen, Germany) running Paravision 5.1, using a 40 mm transreceive coil. Following the acquisition of the anatomical images using the rapid acquisition with the relaxation enhancement (RARE) protocol, diffusion experiments were conducted using the diffusion tensor imaging (DTI) echo planar imaging (DTI-EPI) protocol. The imaging parameters were: slice thickness 0.32 mm, 20 slices, matrix size of 128 × 128 with 0.156 mm × 0.156 mm resolution, δ/Δ = 4/10 ms, 30 directions with b = 670 s/mm^2^ and TE/TR = 23.5/5000 ms. Diffusion images were processed using DSI studio software (http://dsi-studio.labsolver.org, accessed on 4–18 September 2018). The processed data were further analyzed with MATLAB (MathWorks, Natick MA) to obtain fractional anisotropy (FA), radial diffusivity (RD) and axial diffusivity (AD).

### 2.9. Statistical Analysis

All results were expressed as means ± standard error of the mean from at least three independent experiments. Statistical analyses were conducted with the Statistical Package for Social Sciences (SPSS) version 25.0 (IBM Corporation, Armonk, NY, USA). To confirm statistically significant, the one-way ANOVA analysis of variance, followed by a post hoc *LSD* comparison for behavioral assessment and another experiment using Bonferroni comparison, was conducted. A *p*-value less than 0.05 was considered statistically significant.

## 3. Results

### 3.1. In Vivo Expression of OCT4 Improves Behavioral Performance

An initial evaluation was performed before the stereotaxic injection using rotarod and grip strength tests to measure motor coordination and neuromuscular force. Following the treatment, mice were evaluated with the same procedure until terminal stage (12–13 weeks of age) (Figure 1).

**Figure 2:**
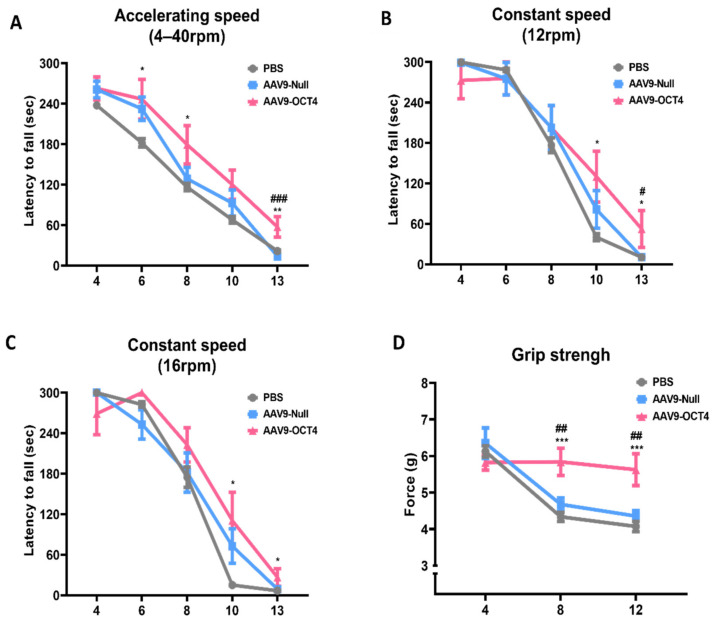
*In vivo* expression of OCT4 improves behavioral performance in HD mice. (**A**) AAV9-OCT4 group displayed significant improvements compared to the control groups (AAV9-Null and PBS) at 6, 8 and 13 weeks of age in the accelerating rotarod test (4–40 rpm) (**B**) at 10 and 13 weeks of age in the constant rotarod test (12 rpm) (**C**) at 10 and 13 weeks of age in the constant rotarod test (16 rpm) and (**D**) at weeks 8 and 12 in the grip strength test. #,* *p* < 0.05, ##,** *p* < 0.01, ***,### *p* < 0.001. Data in all panels represent mean ± SEM. *PBS vs. AAV9-OCT4, #AAV9-Null vs. AAV9-OCT4. PBS group (N = 10); AAV9-Null (N = 13); AAV9-OCT4 (N = 8).

The AAV9-OCT4 group showed a significant increase in latency compared to the control groups in the accelerating speed rotarod test (4–40 rpm) at 6 weeks (PBS = 181.9 ± 19.5, AAV9-Null = 232.4 ± 16.8, AAV9-OCT4 = 246.7 ± 27.6 s), 8 weeks (PBS = 116.4 ± 16.8, AAV9-Null = 128.7 ± 16.2, AAV9-OCT4 = 179.2 ± 26.5 s), 13 weeks of age (PBS = 21.7 ± 4.6, AAV9-Null = 13.9 ± 4.1, AAV9-OCT4 = 57.4 ± 14.2 s) (Figure 2A) and constant speed rotarod test (12 rpm) at 10 weeks (PBS = 40.3 ± 16.2, AAV9-Null = 81.3 ± 28.0, AAV9-OCT4 = 130.1 ± 37.8 s), 13 weeks (PBS = 10.3 ± 2.6, AAV9-Null = 10.7 ± 2.8, AAV9-OCT4 = 52.4.1 ± 27.4 s) (Figure 2B) and in the constant speed rotarod test (16 rpm) at 10 weeks (PBS = 15.1 ± 2.7, AAV9-Null = 72.7 ± 25.5, AAV9-OCT4 = 110.6 ± 42.0 s), 13 weeks (PBS = 6.7 ± 1.6, AAV9-Null = 9.1 ± 1.9, AAV9-OCT4 = 26.4.1 ± 12.8 s) (Figure 2C). In our results, behavioral recovery by OCT4 treatment was observed near 13 weeks of age. The previous study reported that proceeding to late disease stage, which is considered as a humane endpoint, can be acceptable in the form of treatment for these stage [41]. Therefore, we established the endpoint of this study to confirm therapeutic effects.

The AAV9-OCT4 group also displayed significantly increased grip force compared to the control groups and in the grip strength test at 8 weeks (PBS = 4.3 ± 0.3, AAV9-Null = 4.7 ± 0.2, AAV9-OCT4 = 5.8 ± 0.4 g) and 12 weeks of age (PBS = 4.1 ± 0.4, AAV9-Null = 4.4 ± 0.2, AAV9-OCT4 = 5.6 ± 0.4 g) (Figure 2D). These results suggest that OCT4 plays a primary role in improving behavioral performance including motor coordination and neuromuscular force in HD mice.

### 3.2. In Situ Expression of OCT4 Increases NSCs and OPCs in the SVZ

BrdU can track newly proliferated cells. We measured newly proliferated cells during 2 weeks induced by OCT4 via IHC analysis at 6 and 13 weeks of age. A previous study showed that the AAV expression increased most rapidly *in vivo* by 2 weeks following injection of the vector [42]. Therefore, we collected brain tissues for analysis at 2 weeks after transduction. A total of 9 mice (N = 3 per group) at 6 weeks of age were used to assess histologically the ability of OCT4 treatment to elicit the fate of newly proliferated cells in AAV9-OCT4 treated mice relative to PBS and AAV9-Null controls. Then, we evaluated the fate of NSCs or neurons in the SVZ by counting the numbers of cells expressing Nestin^+^BrdU^+^ or βIII-tubulin^+^BrdU^+^. Newly proliferated cells that could possibly differentiate into OPCs or astrocytes were evaluated by counting the numbers of NG2^+^BrdU^+^ or GFAP^+^BrdU^+^ expressing cells. Two weeks after treatment, the fate of NSCs in the SVZ was evaluated through IHC.

**Figure 3:**
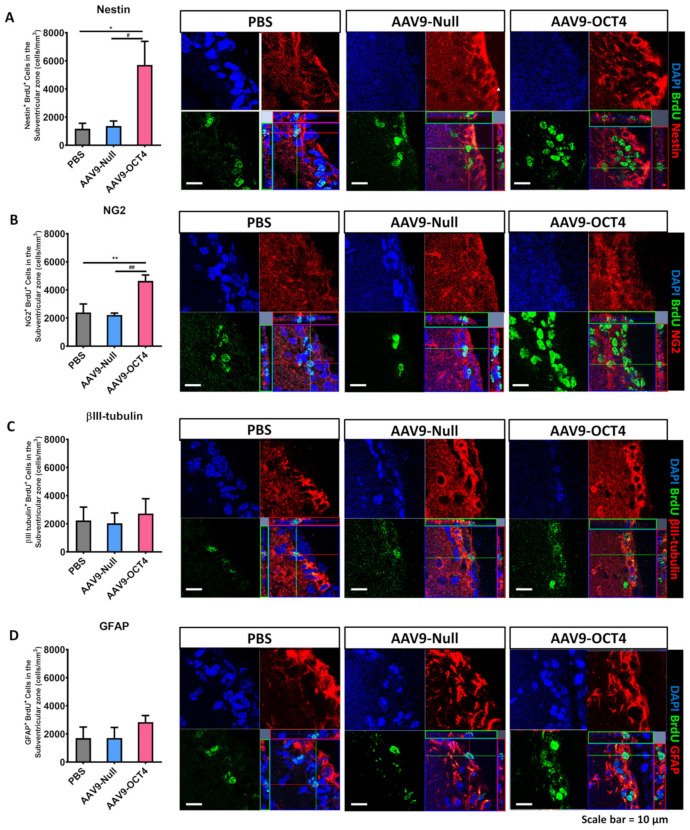
*In situ* expression of OCT4 increases OPCs in the SVZ. To confirm that OPC-related genes proliferation in the SVZ among three groups 2 weeks after stereotaxic injection by using confocal microscopy. (**A**) The numbers of Nestin^+^BrdU^+^ (F = 5.947, * *p* = 0.024; # *p* = 0.041) and (**B**) NG2^+^BrdU^+^ cells were significantly higher in the AAV9-OCT4 group than control groups (F = 9.325, ** *p* = 0.008; ## *p* = 0.006), whereas (**C**) the numbers of βIII-tubulin^+^BrdU^+^ (**D**) and GFAP^+^BrdU^+^ cells did not significantly differ among the three groups. *,# *p* < 0.05, Data in all panels represent mean ± SEM. *PBS vs. AAV9-OCT4, #AAV9-Null vs. AAV9-OCT4. PBS group (N = 3); AAV9-Null (N = 3); AAV9-OCT4 (N = 3).

In the SVZ, the numbers of Nestin^+^BrdU^+^ (PBS = 1.17 ± 0.4, AAV9-Null = 1.4 ± 0.4, AAV9-OCT4 = 5.7 ± 1.8 (×10^3^ cells/mm^3^)) (Figure 3A) and NG2^+^BrdU^+^ (PBS = 2.4 ± 0.7, AAV9-Null = 2.2 ± 0.2, AAV9-OCT4 = 4.6 ± 0.5 (×10^3^ cells/mm^3^)) (Figure 3B) cells were significantly higher in the AAV9-OCT4 group than the controls at 2 weeks post-treatment. The numbers of βIII-tubulin^+^BrdU^+^ (PBS = 2.2 ± 1.0, AAV9-Null = 2.0 ± 0.8, AAV9-OCT4 = 2.7 ± 1.2 (×10^3^ cells/mm^3^)) (Figure 3C) and GFAP^+^BrdU^+^ (PBS = 1.7 ± 0.9, AAV9-Null = 1.7 ± 0.8, AAV9-OCT4 = 2.8 ± 0.5 (×10^3^ cells/mm^3^)) (Figure 3D) cells did not significantly differ among the three groups at 2 weeks post-treatment.

These findings demonstrated that *in situ* expression of OCT4 in the SVZ increases the number of newly generated NSCs and OPCs but not the number of newly generated neurons and astrocytes in HD mice at 2 weeks post-treatment. We tried to check at 2 weeks post-treatment not only how the newly generated cells by OCT4 committed to their fate according to the microenvironment of early-HD stage but also these cells affect early behavior recovery. Because newly proliferated cells in adult concerned behavior at a later time point, more than a month after the injury [43]. We suggest that newly generated NSCs and OPCs by OCT4 affect early behavior recovery at 8 weeks of age.

When HA tag was stained to confirm the transduced regions of AAV9-OCT4 at 6 and 13 weeks of age (N = 3 per group). As expected, HA and OCT4 was extensive overlap in the expression of both epitopes in the transduction region. In addition, control groups not detected HA or OCT4 expression (Figure S1B). Almost HA^+^ cells remain SVZ and CC nearby LV (Figure S2A,B). These results demonstrated that AAV9-OCT4 still remain in the terminal stage. Then we measured the number of transduced cells nearby LV regions (22.1 ± 1.7 × 10^3^ cells/mm^3^, Figure S1C). In addition, we stained HA and Nestin, NG2, βIII-tubulin, and GFAP to confirm the specific cell type of transduced cells. The numbers of NG2^+^HA^+^ cells were higher than Nestin^+^HA^+^ cells, βIII-tubulin^+^HA^+^ cells and GFAP^+^HA^+^ cells in the SVZ (Figure S1C). Therefore, we suggested that AAV-OCT4 transduced NSCs in the SVZ, which in turn can be committed to more number of OPCs within the microenvironmental clues of pre-HD and early-HD stage. Further cell tracking studies are needed to confirm NSC turn to OPCs within microenvironmental clues of pre-HD and early-HD stage.

Therefore, we confirmed cell proliferation by OCT4 at 9 weeks post-treatment and stained the brain tissue with BrdU and Ki67, a cell cycle-associated protein, in the SVZ [44]. However, we rarely found the BrdU^+^ cells in the SVZ at post-treatment 9 weeks (Figure S3A). BrdU can integrate into adult cells undergoing abortive cell cycle entry although these cells then go on to die and disappear within a few weeks [45,46,47,48]. Besides the terminal stage in HD is an insufficient environment for new cells to survive longer. Based on previous studies, the BrdU^+^ newly generated cells initiated by OCT4 during 2 weeks post-treatment period maybe die at the terminal stage of HD. Therefore, BrdU^+^ cells could hardly be seen at 13 weeks of age.

Instead of BrdU staining, we stained with Ki67 for the evaluation of cell proliferation at 13 weeks of age (Figure S3B). As a result, cell proliferation at the terminal stage was significantly increased in the AAV9-OCT4 group compared to the control group.

Taken together, our results suggest that the *in situ* expression of OCT4 can induce the NSC proliferation in the SVZ during OCT4 treatment initial 2 weeks, which in turn can be committed to more number of OPCs within the microenvironmental clues of pre-HD stage. Unfortunately, BrdU positive cells by OCT4 during initial 2 weeks did not survive until the terminal stage. However, the continuous expression of AAV9-OCT4 at 9 weeks post-treatment may influence cell proliferation at the terminal stage.

### 3.3. OCT4-Induced OPCs Ameliorate Myelination Deficits of HD Mice

**Figure 4:**
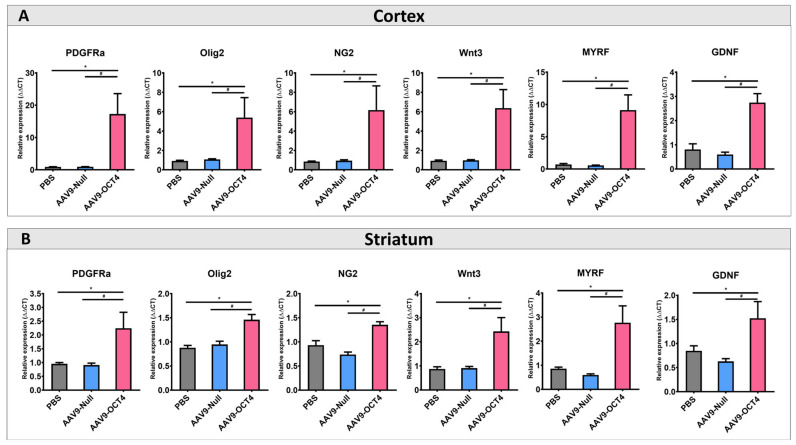
*In situ* expression of OCT4 increases OPC-related genes. (**A**) To confirm that OPC-related genes proliferation in the cortex (**B**) and striatum among three groups at 13 weeks of age by using qRT-PCR. AAV9-OCT4 group significantly increased expression levels of NG2, Olig2, PDGFRa, Wnt3, MYRF and GDNF in the cortex and striatum *,# *p* < 0.05, Data in all panels represent mean ± SEM. * PBS vs. AAV9-OCT4, # AAV9-Null vs. AAV9-OCT4. PBS group (N = 4); AAV9-Null (N = 4); AAV9-OCT4 (N = 3).

Next, we confirmed the effects of OCT4 on OPC-related gene expression specific to the microenvironment in late-HD stage. The expressions of OPC-related markers NG2, Olig2, PDGFRα, Wnt3, MYRF and GDNF were confirmed by qRT-PCR. The AAV9-OCT4 group displayed significantly increased the expression levels of OPC-related markers in the cortex (Figure 4A) and striatum (Figure 4B) at 13 weeks of age. NSCs and OPCs secrete various growth factors, neurotrophic factors, and cytokines, thus protecting existing neural cells against damage *in situ* [49]. Therefore, these results suggest that the secreated factors such as MYRF and GDNF from the activated NSCs and OPCs by OCT4 induced functional recovery at the terminal stage.

**Figure 5:**
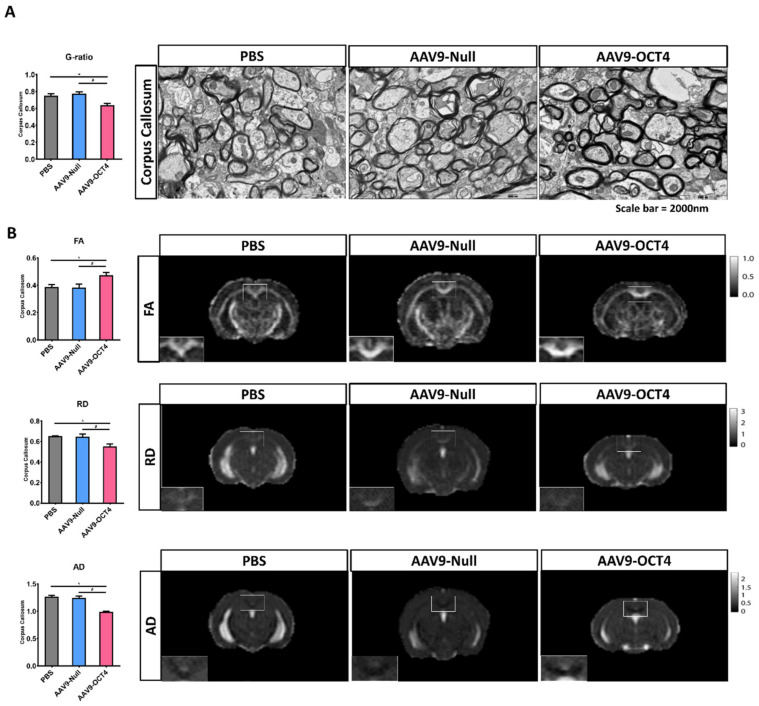
OCT4-induced OPCs ameliorate myelination deficits (**A**) TEM was used to visualize myelinated fibers in corpus callosum at 13 weeks of age, the value of g-ratio in the AAV9-OCT4 group was significantly lower than control groups (F = 11.442, * *p* = 0.001; # *p* < 0.001). (**B**) MRI results were analyzed for FA (F = 5.986, * *p* = 0.049; # *p* = 0.042), RD (F = 8.040, * *p* = 0.018; # *p* = 0.026) and AD (F = 34.309, * *p* = 0.015; # *p* = 0.019), myelination defects in the AAV9-OCT4 group was significantly ameliorated. *,# *p* < 0.05, Data in all panels represent mean ± SEM. * PBS vs. AAV9-OCT4, #AAV9-Null vs. AAV9-OCT4. PBS group (N = 3); AAV9-Null (N = 3); AAV9-OCT4 (N = 3).

TEM and MRI were used to visualize myelinated fibers in CC, the largest white matter structure in the brain, at 13 weeks of age. AAV9-OCT4 group presented slightly decreased g-ratio (the numerical ratio of the axonal diameter divided by the diameter of the myelinated axons) compared with control groups (Figure 5A). This g-ratio analysis via TEM images result indicated that AAV9-OCT4 myelin sheaths were thicker than those of control groups (PBS = 0.75 ± 0.02, AAV9-Null = 0.78 ± 0.02, AAV9-OCT4 = 0.64 ± 0.02).

The FA value increased in CC of AAV9-OCT4 group, suggesting the increased structural integrity of this fiber tract (PBS = 0.39 ± 0.02, AAV9-Null = 0.38 ± 0.03, AAV9-OCT4 = 0.47 ± 0.04). RD (PBS = 0.65 ± 0.02, AAV9-Null = 0.65 ± 0.03, AAV9-OCT4 = 0.55 ± 0.04) and AD (PBS = 1.27 ± 0.03, AAV9-Null = 1.25 ± 0.05, AAV9-OCT4 = 0.99 ± 0.06) values reduced in CC of AAV9-OCT4 group. These results implicated that demyelination was attenuated in AAV9-OCT4 group.

Taken together, TEM and MRI results were analyzed to confirm that myelination defects were significantly reduced in the AAV9-OCT4 group (Figure 5B). These results suggested that OCT4 overexpression induces myelin plasticity via the activation of OPC-related genes and ameliorates myelination deficits of HD mice.

### 3.4. Subependymal OCT4 Expression Induces Striatal Neuroprotection

**Figure 6:**
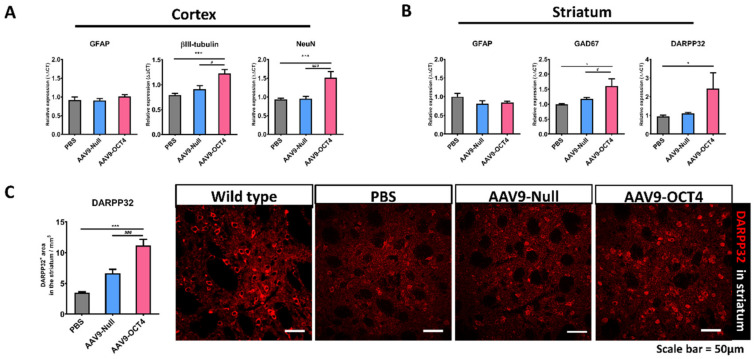
OCT4-induced OPCs induce neuroprotection. (**A**) To confirm neuropretection in the cortex (**B**) and striatum among three groups at 13 weeks of age by qRT-PCR. AAV9-OCT4 group displayed significantly increased expression of βIII tubulin (F = 12.271, *** *p* < 0.001; # *p* = 0.004), NeuN (F = 13.477, *** *p* < 0.001; ### *p* < 0.001), a neuronal marker in the cortex and GAD67 (F = 8.057, * *p* = 0.001; # *p* = 0.033) and DARPP32(F = 3.794, * *p* = 0.047), a GABAergic neuronal marker in the striatum. However, the AAV9-OCT4 group did not change the expression of GFAP, an astrocytic marker in both regions (**C**) In the striatum, AAV9-OCT4 group significantly increased the area of DARPP32^+^ GABAergic neurons compared to controls (F = 18.697, *** *p* < 0.001; ### *p* < 0.001). *,# *p* < 0.05, ***,### *p* < 0.001, Data in all panels represent mean ± SEM. *PBS vs. AAV9-OCT4, #AAV9-Null vs. AAV9-OCT4. PBS group (N = 4); AAV9-Null (N = 4); AAV9-OCT4 (N = 3).

The expression of βIII-tubulin, NeuN, glutamic acid decarboxylase 67 (GAD67), DARPP32 and GFAP was confirmed by qRT-PCR at 13 weeks of age. AAV9-OCT4 group displayed significantly increased not only expression of βIII-tubulin and NeuN, a neuronal marker in the cortex but also GAD67 and DARPP32, a GABAergic neuronal marker in the striatum. However, the AAV9-OCT4 group did not change the expression of GFAP, an astrocytic marker in both regions (Figure 6A,B). In addition, DARPP-32^+^ GABAergic neurons of the striatal region significantly increased in AAV9-OCT4 group compared to control groups (PBS = 3.48 ± 0.16, AAV9-Null = 6.68 ± 0.62, AAV9-OCT4 = 11.17 ± 1.00) using confocal microscopy (Figure 6C). OPCs have been shown to express various growth factors and cytokines that play a significant role in cell functions and survival [50,51]. Previous studies reported that GDNF, mainly released by OPCs and oligodendrocytes, can promote neuronal cell survival as well as axon regeneration and myelination in demyelinating conditions [50,52]. Taken together, these results raise the potential that OCT4 overexpression not only ameliorates myelination deficits but also induces striatal neuroprotection in HD.

## 4. Discussion

Recent studies have reported that WM is associated with motor and cognitive functions, which explains why HD-induced WM atrophy causes the behavioral defects [5,53]. Especially, demyelination induced neuronal firing rate abnormalities was shown in the motor cortex, as well as deficits in motor performance [54]. The pre-HD stage of R6/2 mice shows myelin deficits in the CC at 2 weeks of age [55] before the remarkable GABAergic neuronal loss, which is known to occur at late-HD stage.

In this study, we performed accelerating speed (4–40 rpm) and constant speeds (12, 16 rpm) in the rotarod test for motor coordination. A rotarod test with accelerating speed is usually used to confirm the efficacy of the treatment. On the other hand, a rotarod test with appropriate fixed speed can sensitively represent the difference as to distinguish between the control group and the treatment group relative to the accelerating test [56,57,58]. Therefore, we tried to perform both accelerating speed and constant speeds in the rotarod test for delicate measures of motor coordination. We also used grip strength test to measure neuromuscular force. The pathophysiological and behavioral phenotype of R6/2 mice came to be prominent at 8 weeks of age. In this age, R6/2 mice had more severely deteriorated motor coordination than deteriorated neuromuscular force [36].

Rotarod test is used to investigate motor coordination and balance and grip strength test is used to assess neuromuscular strength. In our results, the AAV9-OCT4 group slightly improved in the rotarod at 6–8 weeks of age and the grip strength significant improved at 8 weeks of age (Figure 2A–D). However, the HD animal model, R6/2, has many CAG repeats (approximately 160 ± 5 CAG repeats), and this CAG repeat can induce serious movement problems including motor coordination problem over time. Therefore, we suggested that the significant functional improvement by the therapeutic effects of OCT4 may appear later in motor coordination than grip strength.

Taken together, following *in vivo* expression of reprogramming factor OCT4 in pre-HD R6/2 mice, the rotarod and grip strength tests revealed significant motor improvements in the late-HD stage of OCT4 overexpressing mice compared to the control groups, suggesting that OCT4 may be the primary role in improving behavioral performance in HD mice. The behavioral improvement in AAV9-OCT4 group at terminal stage was shown in recorded videos in Figure S4.

We then observed that OCT4 using AAV9 vector administrated into the LV have an effect on the NSC niche activation in the SVZ. AAV is one of the most common vectors used in gene therapy [59]. AAV vectors have an excellent safety record in clinical trials and preclinical animal studies [60,61]. AAV as a gene delivery has benefits and multiple natural serotypes such as serotype 9 which can infect target cells in various tissues including brain. Although mild immune responses can occur when AAV infects target cells, AAV is considered as the material with biosafety compared to another popular viral vector. Therefore, we used AAV9 for the safety and for the transduction efficacy as a brain viral vector [62,63,64,65]. Our data showed that AAV9-OCT4 expression remain in the terminal stage near LV region (Figure S2A,B). Then, we performed stereology-based quantification of the percentages of transduced cells after LV delivery of AAV9 (1 × 10^12^ vg/mL, 1 μL each LV, N = 3). Previous studies reported that transduction efficacy of AAV9 in adulthood depend on injection route and titer [66,67]. Therefore, we suggested the low transduction efficacy of AAV9-OCT4 due to the LV route delivery and low concentration. Further dose-escalation studies are needed to confirm the therapeutic and pathophysiological effects of OCT4 based on the differential concentrations, from low dose to high dose.

Although our result demonstrated low transduction efficacy of AAV9-OCT4, we investigated that the effects of *in vivo* expression of OCT4 on NSC niche activation in the SVZ and induction of cell fate specific to the changed microenvironment of pre-HD stage through BrdU labeling (Figure 3). Although the proliferation cells with BrdU labeling by OCT4 treatment during 2 weeks post-treatment disappeared at the terminal stage (Figure S3A), the continuous expression of AAV9-OCT4 at 9 weeks post-treatment (Figure S1B) may influence cell proliferation (Figure S3B), though it was not specified that which kind of cells turn toward OPCs. Therefore, we need to conduct a further study with a tissue or cell type specific promotor such as nestin promotor or oligodendrocyte promotor.

The SVZ contains the largest niche areas for newly generated neural cells in the adult brain [68,69,70,71]. In the damaged WM areas, the therapeutic mechanism to induce endogenous NSC activation in the SVZ can change the fate of NSCs into oligodendrocyte-lineage cells in order to compensate for myelin deficits [71]. We tried to investigate how the newly generated cells committed to their fate according to the microenvironment at post-treatment 2 weeks. The AAV mediated gene expression increases progressively starting from the day after vector administration. It reached at the maximum expression at 2 weeks after the injection [42]. Therefore, we confirmed IHC analysis at 6 weeks of age. Namely, *in situ* expression of OCT4 in the SVZ increased the number of newly generated NSCs (Nestin^+^BrdU^+^ cells) and OPCs (NG2^+^BrdU^+^ cells) but not the number of newly generated neurons (βIII-tubulin^+^BrdU^+^ cells) and astrocytes (GFAP^+^BrdU^+^ cells) in HD mice at 6 weeks of age (Figure 2A–D). Newly proliferated cells in adult affected behavior at a later time point, more than a month after injury [43]. Therefore, we suggest that the increased number of NSCs and OPCs during initial 2 weeks affected the recovery of neuromuscular function at 8 weeks of age.

When we stained the brain tissue with BrdU in SVZ, our data displayed that BrdU^+^ cells were rarely found in the SVZ at post-treatment 9 weeks (Figure S3B). Although BrdU can integrate into adult cells undergoing abortive cell cycle entry, most cells then go on to die and disappear within a few weeks [45,46,47,48]. Therefore, newly generated cells initiated by OCT4 during 2 weeks post-treatment might die at the terminal stage of HD, and BrdU^+^ cells could hardly be seen at 13 weeks of age. Instead of BrdU staining, when we stained with Ki67, a cell cycle-associated protein, in SVZ [44] for the evaluation of cell proliferation at 13 weeks of age (Figure S3C), cell proliferation of the OCT4 group significantly increased compared to the control group.

Brain damages induced by the conditions of neurodegenerative diseases continuously activated the proliferation of neural stem/progenitor cells in the SVZ. Ki67 is present during every phase of the cell cycle in asynchronously cycling cells and absent in non-dividing cells [72,73]. Therefore, Ki67-labeled cells in the late-HD stage didn’t distinguish between the brain damage-derived from proliferated cells and the newly generated cells by OCT4. However, we injected BrdU after OCT4 treatment during 2 weeks. Therefore, BrdU^+^ cells by injection were the newly generated cells after OCT4 treatment during 2 weeks. In order to overcome this limitation, it is necessary to inject BrdU for longer period of time to label newly generated cells throughout the whole experimental period in further study.

Next, we confirmed the effects of OCT4 on OPC-related gene expression specific to the microenvironment in HD at 13 weeks of age. The OCT4-induced OPCs enhanced myelin plasticity via the activation of OPC-related genes (Figure 4A,B). NG2 and Olig2 are used to specifically identify OPCs [74]. Olig2, PDGFRa and Wnt3 induce the differentiation of OPCs to oligodendrocytes [75,76,77]. Particularly, our results displayed that OCT4-induced OPCs upregulated MYRF expression. Since HD causes MYRF downregulation and leads to oligodendrocyte death and demyelination [78], OCT4-mediated MYRF upregulation can ameliorate myelination deficits in the WM of brains with HD. In addition, OPCs have been shown to express various growth factors and cytokines that play a significant role in cell functions and survival [50,51]. Previous studies reported that GDNF, mainly released by OPCs and oligodendrocytes, can promote neuronal cell survival as well as axon regeneration and myelination in demyelinating conditions [50,52]. The neuroprotective functions of secreted GDNF also could benefit GABAergic neurons in the striatum and mitigate the pathophysiological effects of HD, all of which has been reported to improve behavioral functions.

Our data showed that OCT4-induced proliferation of NSCs and OPCs at 6 weeks of age and expression of OPC-related genes increased at 13 weeks of age. Previous study reported that NSCs and OPCs secrete various growth factors including neurotrophic factors, growth factors and cytokines, thus protecting pre-existing neural cells against damage *in situ* [39]. Therefore, our results suggest that the transduced cells in the SVZ and CC may affect the nearby brain regions such as cerebral cortex and striatum through paracrine effects by the upregulation of MYRF and GDNF secreted from the activated NSCs and OPCs by OCT4, consequently inducing therapeutic effects of modest neuroprotection and functional recovery at the terminal stage of HD.

Our results suggest that the *in situ* expression of OCT4 can induce the NSC proliferation in the SVZ, which in turn can be committed to more number of OPCs within the microenvironmental clues of pre-HD and early-HD stage. The increased number of initially proliferated NSCs and OPCs affected behavioral recovery after OCT4 treatment. However, these cells did not remain at the terminal stage.

For the analysis of health axon with myelin in CC via analysis of TEM images, g-ratio was calculated in each axon with myelin. The values of g-ratio of AAV9-OCT4 group were significantly lower than those in control groups, indicating that OCT4 overexpression ameliorated myelination deficits of HD mice (Figure 5A). It is important to note that FA is positively correlated to the myelination and organization of WM fibers [79], and early increase in RD and AD are followed by significant changes in neuronal volume loss in HD [80,81,82]. Our data showed that AAV9-OCT4 group had significantly higher FA and lower RD, and AD compared to control groups (Figure 5B). In addition, OCT4 overexpression also protected mature neurons, not astrocytes, in the cortex and striatum (Figure 6A). Particularly, the expression of striatal DARPP32^+^ GABAergic neurons significantly increased in the AAV9-OCT4 group compared to control groups (Figure 6B). Subependymal cells were transduced in the SVZ along with intracerevroventricular injection until the terminal stage (Figure S2A). This result suggests that OCT4 overexpression continuously affected cell proliferation in SVZ. Although the proliferated cells disappeared during first few weeks, these cells can secrete various growth factors such as GDNF for protecting pre-existing neural cells against the damage. However, in this study, the therapeutic mechanism of secreted factors by OCT4-derived proliferated cells influenced neuroprotection was not thoroughly investigated. Therefore, a further study to uncover this therapeutic mechanism is needed.

In summary, our data demonstrated that *in vivo* reprogramming of OCT4 at pre-HD stage can ameliorate myelination deficits and induce neuroprotection by the secreted growth factors such as MYRF and GDNF from activated NSC and OPCs. This neuroprotection, in turn, lead to the behavioral recovery in HD mice.

## 5. Conclusions

**Figure 7:**
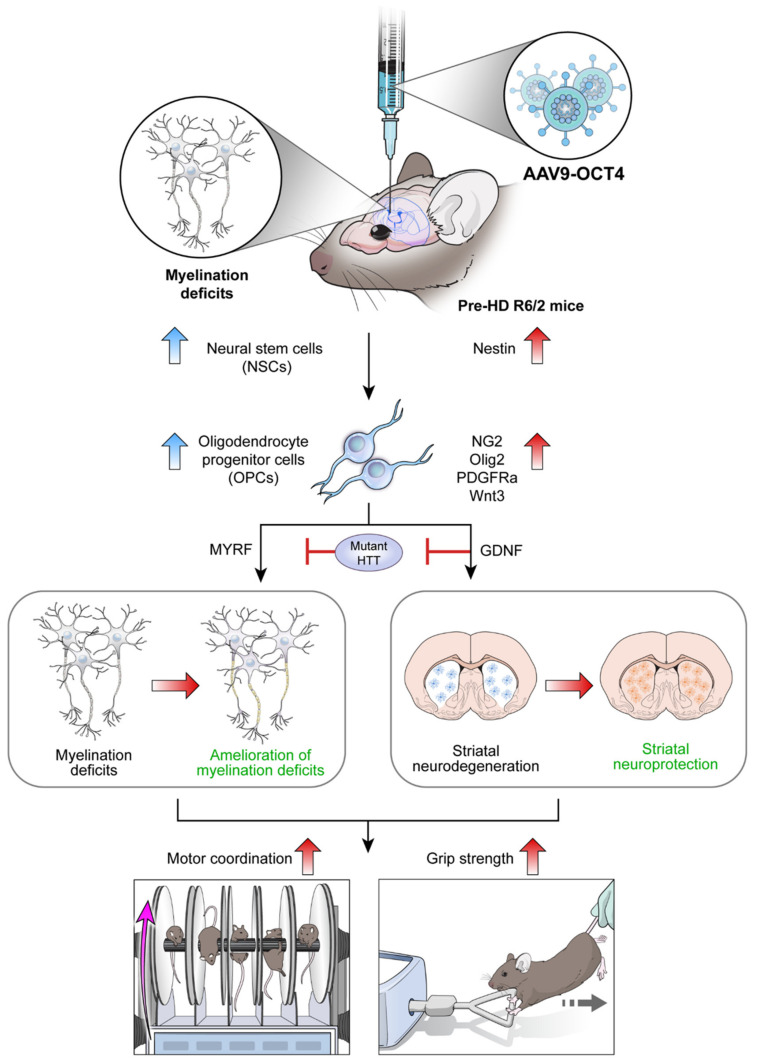
Scheme of *in vivo* expression of reprogramming factor OCT4 in R6/2 HD mice. Myelin deficits emerge in the corpus callosum at 4 weeks of age, pre-symptomatic (pre-HD) stage before remarkable GABAergic neuronal loss at late-symptomatic (late-HD) stage in R6/2 mice. *In situ* expression of reprogramming factor OCT4 induces NSC niche activation in the SVZ and changes cell fate specific to the microenvironment of HD from NSCs to OPCs. Particularly, MYRF and GDNF released by OCT4-induced OPCs seem to ameliorate myelination deficits and induce striatal neuroprotection, consequently improving behavioral performances such as motor coordination and grip strength in HD.

*In situ* expression of reprogramming factor OCT4 induces NSC niche activation in the SVZ and changes cell fate specific to the microenvironment of HD from NSCs to OPCs. Particularly, MYRF and GDNF released by OPCs seem to ameliorate myelination deficits and induce striatal neuroprotection in HD, which explains the behavioral improvement such as motor coordination and grip strength in R6/2 mice overexpressing OCT4 (Figure 7). Taken together, these results raise the potential that OCT4-induced OPCs not only ameliorates myelination deficits but also induces striatal neuroprotection in HD.

## Data Availability

Data is contained within the article or Supplementary Material. The data presented in this study are available.

## Supplementary Materials

The following are available online at https://www.mdpi.com/article/10.3390/genes12050712/s1, Figure S1: Characteristics and phenotype of AAV9-OCT4 transduced cells, Figure S2: AAV9-OCT4 transduction in subventricular zone and corpus callosum, Figure S3: Cell proliferation by AAV9-OCT4, Figure S4: Assessment of locomotor activity and rearing behavior.
