# Supplementary material for: In Vivo Expression of Reprogramming Factor OCT4 Ameliorates Myelination Deficits and Induces Striatal Neuroprotection in Huntington’s Disease
Source: Genes (Basel). 2021 May 10;12(5):712. doi: 10.3390/genes12050712 (PMC8150572; doi:10.3390/genes12050712)
Supplement: Supplementary file 1 [file genes-12-00712-s001.zip › 210427 supplement 1~3.pdf]

Supplementary figure 1

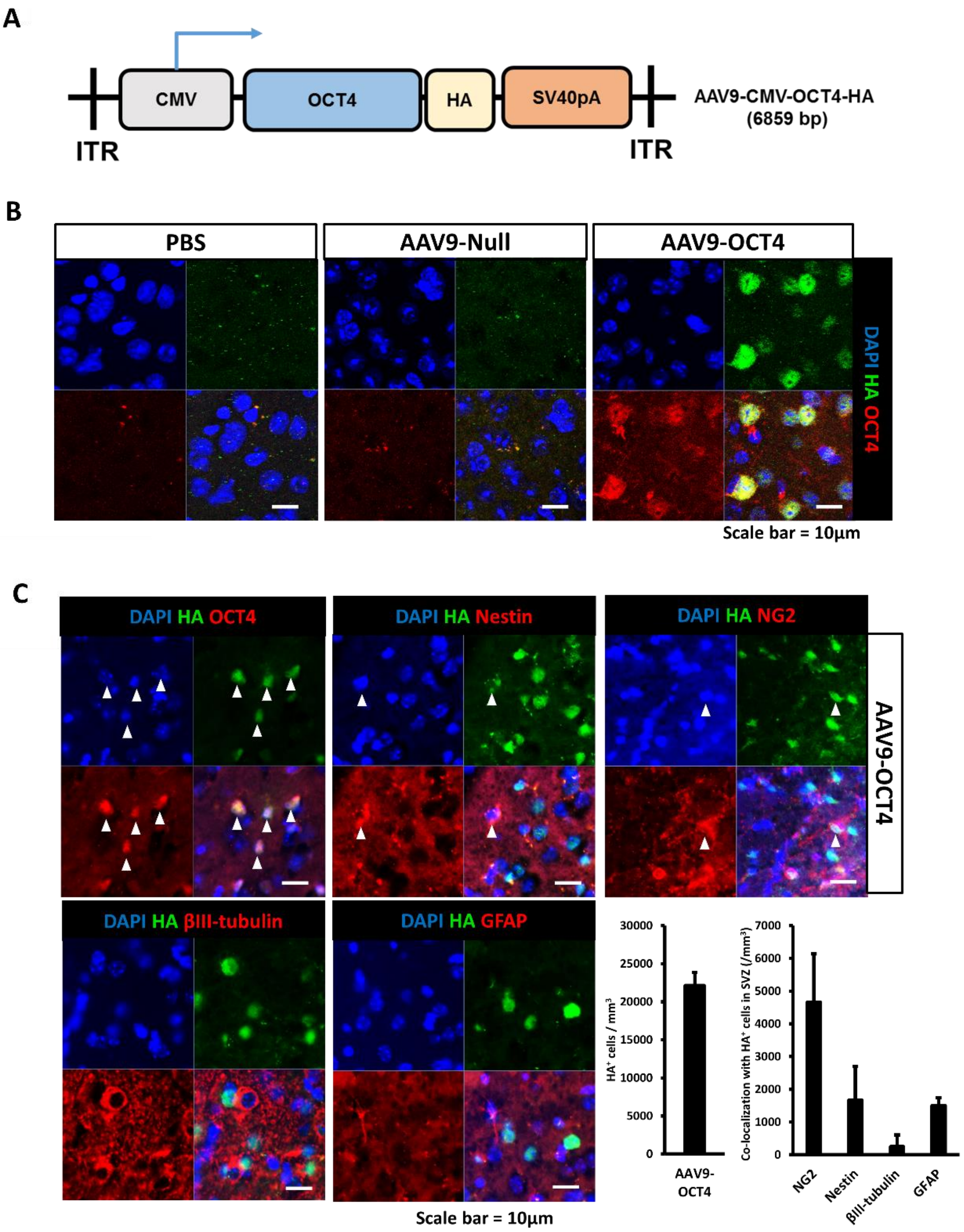

Figure S1. Characteristics and phenotype of AAV9-OCT4 transduced cells. (A) schematic representation of the AAV9-OCT4 vector. (B) Representative images of HA tag and OCT4 expression in the transduction region at 13 weeks of age. AAV9-OCT4 group only detected HA and OCT4 co-localization. Scale bar = 10 μm. (C) The number of HA+ cells (/mm3) and representative images of HA tag with OCT4, Nestin, NG2, βIII-tubulin and GFAP expression at 6 weeks of age. The numbers of $NG2^{+}HA^{+}$ cells were higher than $Nestin^{+}HA^{+}$ cells, βIII-$tubulin^{+}HA^{+}$ cells and $GFAP^{+}HA^{+}$ cells in the SVZ. Scale bar = 10 μm.

Supplementary figure 2

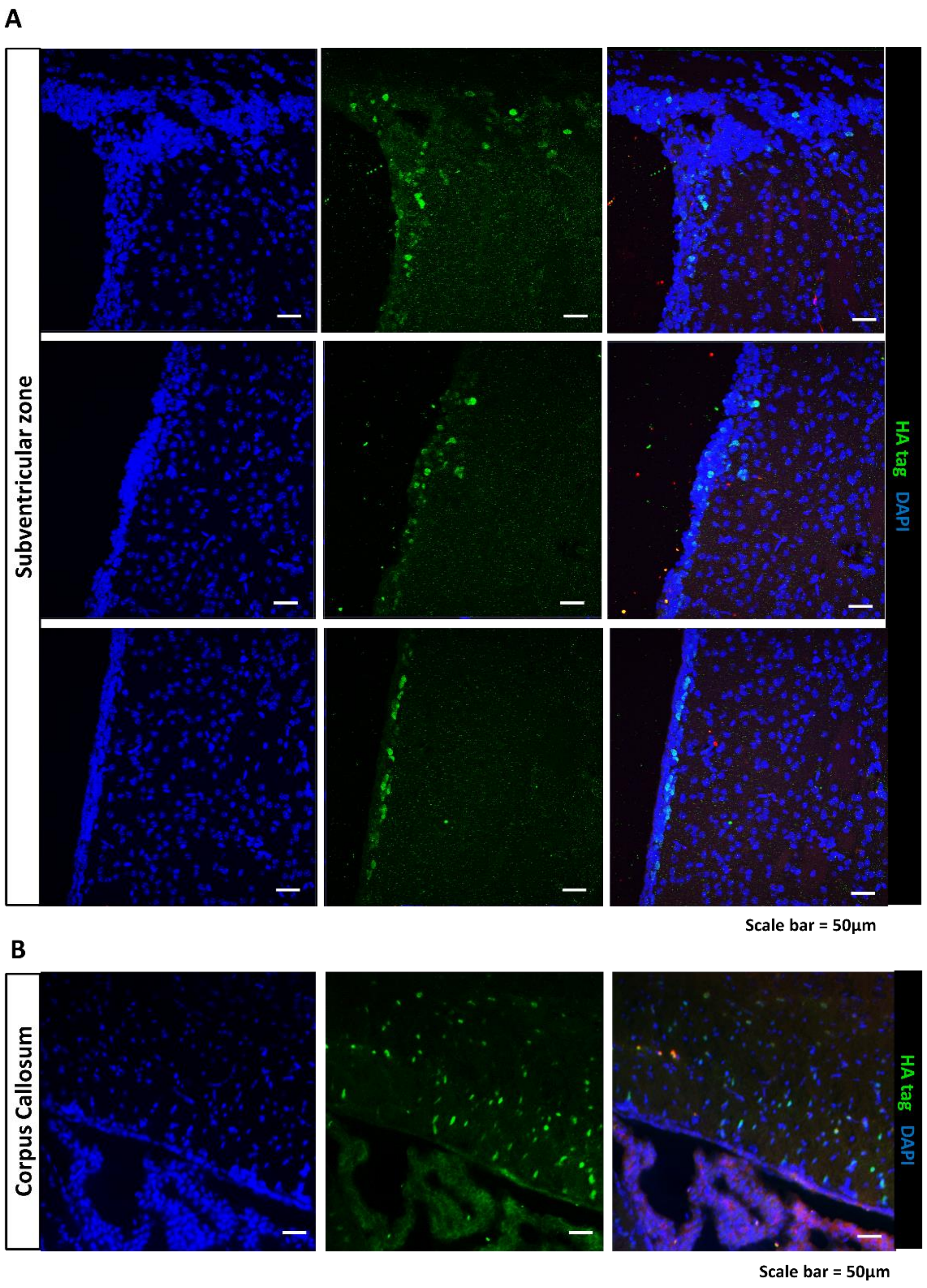

Figure S2. AAV9-OCT4 transduction in subventricular zone and corpus callosum. When the AAV9-OCT4 transduction was confirmed in the transduced regions of AAV9-OCT4 group (N=3), the number of HA^+ cells remained until 13 weeks of age. (A) Representative images of HA tag in the subventricular zone at 13 weeks of age. (B) Representative images of HA tag in the corpus callosum at 13 weeks of age. Scale bar = 50 μm.

Supplementary figure 3

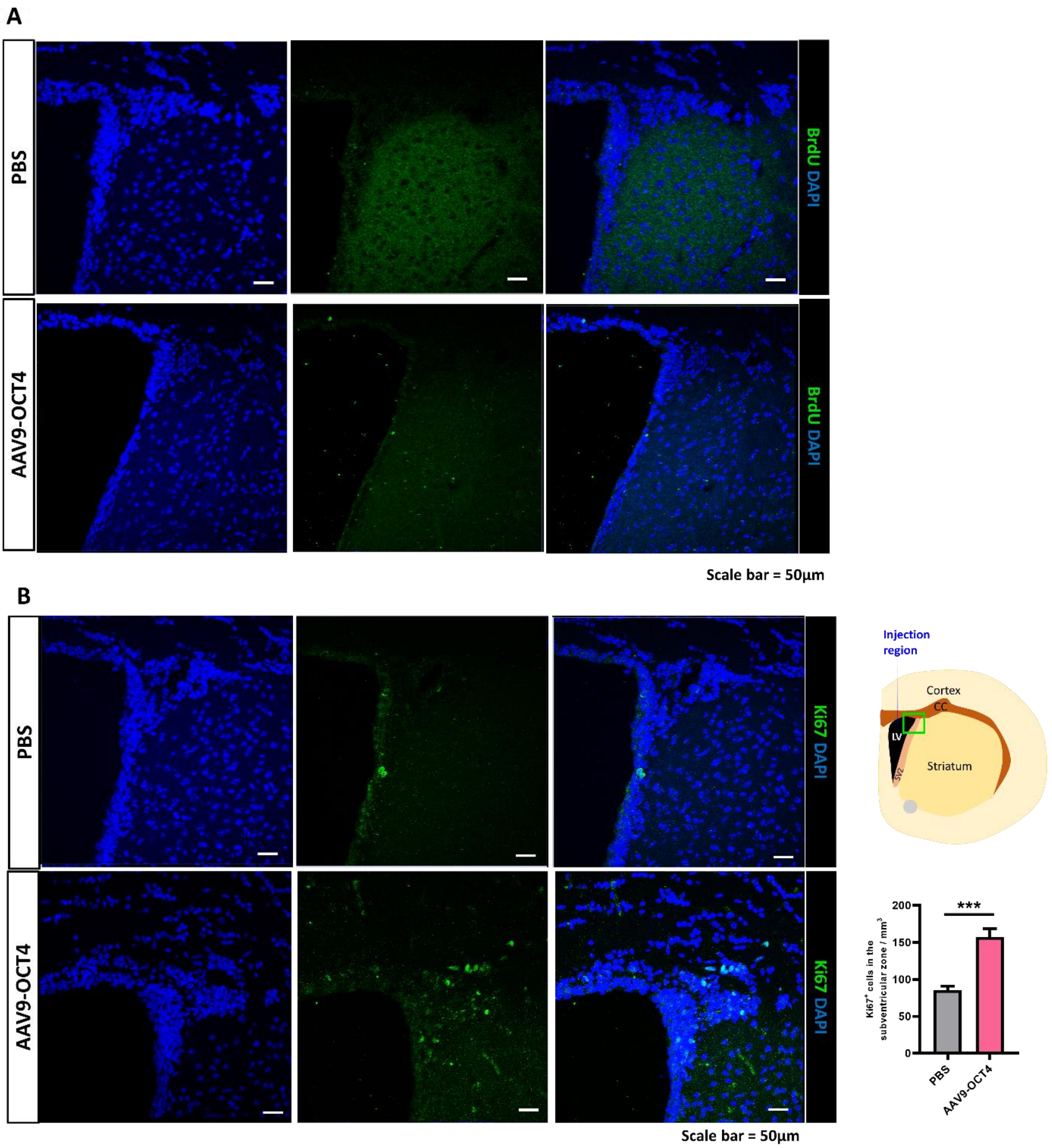

Figure S3. Cell proliferation by AAV9-OCT4. (A) The brain tissue was stained with BrdU, our data displayed that BrdU$^+$ cells were rarely found in the SVZ at post-treatment 9 weeks. PBS group (N=3); AAV9-OCT4 group (N=3). (C) When Ki67, a cell cycle-associated protein, was stained for the evaluation of cell proliferation at 13 weeks of age, cell proliferation in the SVZ at the terminal stage significantly increased compared to untreated transgenic mice (PBS = 85.6±10.9, AAV9-OCT4 = 157.5±29.4 (cells/mm^3), F=2.455, ***p=0.001). independent t-test, PBS group (N=3); AAV9-OCT4 group (N=3).
