# Supplementary material for: In Vivo Expression of Reprogramming Factor OCT4 Ameliorates Myelination Deficits and Induces Striatal Neuroprotection in Huntington’s Disease
Source: Genes (Basel). 2021 May 10;12(5):712. doi: 10.3390/genes12050712 (PMC8150572; doi:10.3390/genes12050712)
Supplement: Supplementary file 1 [file genes-12-00712-s001.zip › 210427 supplement 4.pptx]

## Slide 1
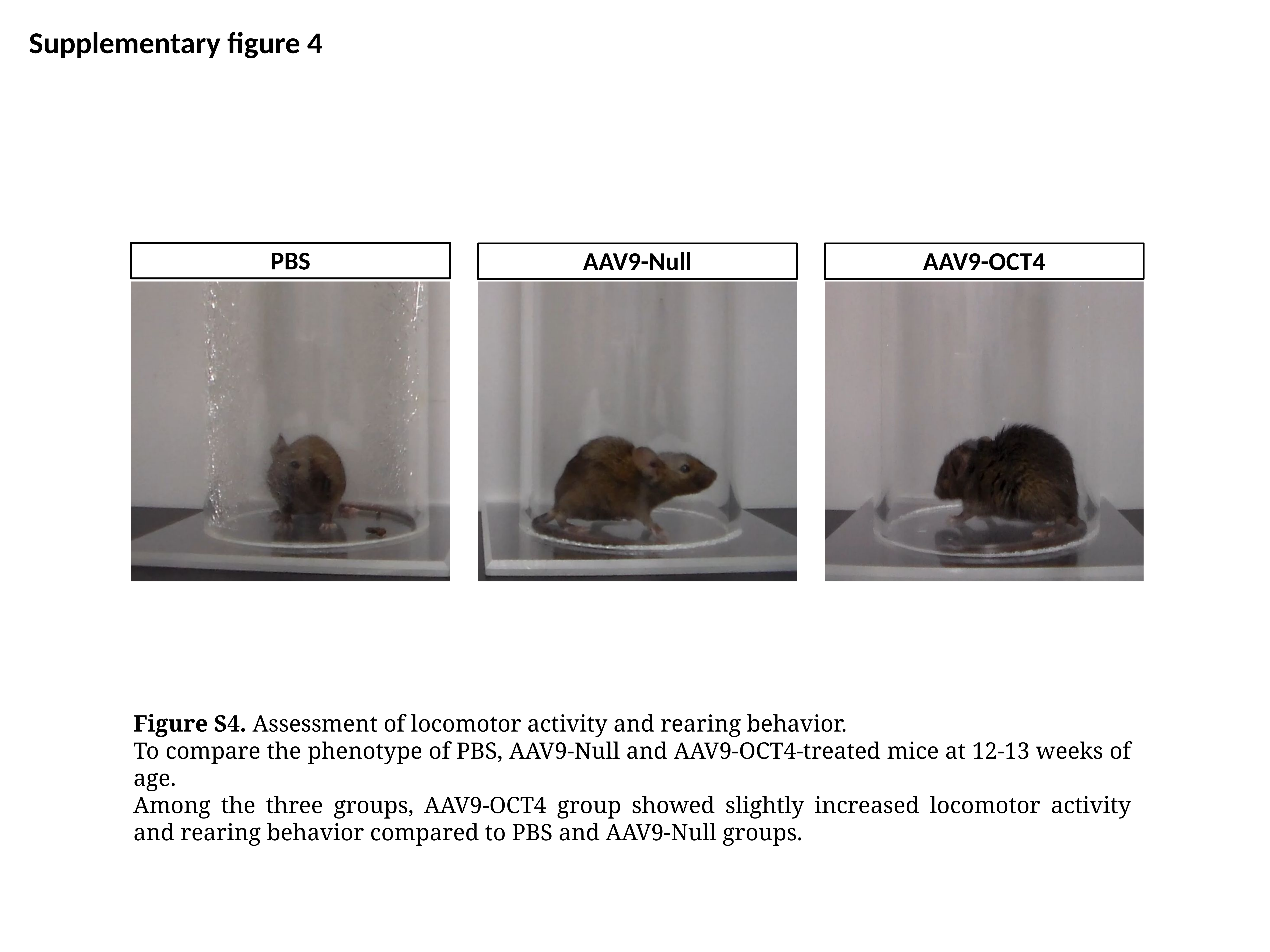

Supplementary figure 4
PBS
AAV9-Null
AAV9-OCT4
Figure S4. Assessment of locomotor activity and rearing behavior.
To compare the phenotype of PBS, AAV9-Null and AAV9-OCT4-treated mice at 12-13 weeks of age.
Among the three groups, AAV9-OCT4 group showed slightly increased locomotor activity and rearing behavior compared to PBS and AAV9-Null groups.
